# Supplementary material for: Maths attitudes, school affect and teacher characteristics as predictors of maths attainment trajectories in primary and secondary education
Source: R Soc Open Sci. 2020 Oct 7;7(10):200975. doi: 10.1098/rsos.200975 (PMC7657886; doi:10.1098/rsos.200975)
Supplement: Items for composite measures [file rsos200975supp1.docx]

**Supplemental Material – RSOS-200975**

| **Composite measure** | **Items** |
| --- | --- |
| School affect (P) | My school was a place where other pupils accepted me as I am  My school was a place where I felt lonely  My school was a place where I felt worried  My school was a place where I people trusted me  My school was a place where I knew people thought a lot of me  My school was a place where I got on well with the other pupils in my class  My school was a place where other pupils were very friendly  My school was a place where I felt restless |
| S-T relationships (P) | My school was a place where I felt proud to be a pupil  My school was a place where my teacher listens to what I say  My school was a place where my teacher treated me fairly in class |
| Maths attitudes (P) | I hate mathematics  Maths is easy for me  I look forward to mathematics  I get good marks in maths  I am interested in maths  I learn things quickly in maths  I like maths  I’m good at maths  I enjoy doing work in maths  I am bad at maths |
| Teacher affect (P) | I really enjoy teaching  I would prefer to get out of teaching  I like the challenge of making children understand  I really enjoy teaching numeracy skills  Changes in the curriculum are an exciting challenge  Being a teacher is really worthwhile |
| School belonging (S) | My school is a place where other pupils accept me as I am  My school is a place where I feel proud to be a pupil  My school is a place where people trust me  My school is a place where I know people think a lot of me  My school is a place where I get on well with other pupils in my classes  My school is a place where other pupils are very friendly |
| S-T relationships (S) | Most of my teachers don't really listen to what I say in class  I get treated unfairly by most of my teachers |
| Negative school emotion (S) | My school is a place where I feel lonely  My school is a place where I feel worried  My school is a place where I feel restless |
| Maths attitudes (S) | How much do you like doing maths?  In general, how useful is what you learn in maths?  For me, being good in these subjects (maths) is (important) |
| Positive teaching (S) | My maths teacher tries to make maths interesting  My maths teacher likes maths  My maths teacher understands maths really well  My maths teacher can explain things to me when I don’t understand them  My maths teacher has helped me learn things in maths that I thought I couldn’t understand  Everyone is encouraged to do their very best  My maths teacher cares about how we feel about life in general  My maths teacher is friendly to us  My maths teacher criticises all of us equally if we do poor work  My maths teacher gives us time to really explore and understand new things  In our class, trying hard is very important |
| Teacher fairness (S) | My maths teacher only cares about the clever students  My maths teacher has given up on some of the students in the class  My maths teacher thinks that some of the students in this class can’t do very good work  My maths teacher treats boys and girls differently  My maths teacher treats some students better than other students |

Note. P = measured in primary education, S = measured in secondary education.
